# CIN2 in the Era of Risk-Based Management and HPV Vaccination: Epidemiology, Natural History and Guidelines

**DOI:** 10.3390/diagnostics15192512

**Published:** 2025-10-02

**Authors:** Maria Teresa Bruno, Alessia Pagana, Carla Lo Giudice, Marco Marzio Panella, Giuseppe Mascellino, Antonio Simone Laganà

**Affiliations:** 1 Department of General Surgery and Medical Surgery Specialties, Gynecological Clinic, University of Catania, 95123 Catania, Italy; alessia.pagana@studium.unict.it (A.P.); carla.logiudice@studium.unict.it (C.L.G.);; 2 Unit of Obstetrics and Gynecology, “Paolo Giaccone” Hospital, Department of Health Promotion, Mother and Child Care, Internal Medicine and Medical Specialties (PROMISE), University of Palermo, 90127 Palermo, Italy

**Keywords:** CIN2, CIN2+, active surveillance, guidelines, CIN2 regression, risk-based management, HPV vaccination

## Abstract

**Background:** Cervical intraepithelial neoplasia grade 2 (CIN2) represents a controversial lesion in cervical cancer prevention. Traditionally included in the aggregate CIN2+ endpoint for reasons of diagnostic stability and statistical power, isolated CIN2 has unique biological characteristics: greater interobserver variability, a high probability of spontaneous regression and a lower risk of progression compared to CIN3. **Objectives:** To critically describe the epidemiology, natural history and management strategies of CIN2, integrating data from clinical and population-based studies and comparing the recommendations of the main international guidelines. **Methods:** A narrative review was conducted using a search of PubMed and Scopus (1990–January 2025). Prospective and retrospective studies on isolated CIN2, screening and vaccination trials with CIN2+ endpoints, biomarker research, and consensus documents (ASCCP, ESGO, GISCi, Ministry of Health, WHO) were included. **Results:** Clinical studies have shown a high probability of CIN2 regression (50–70% within two years, >70% in those <25 years), compared to a 10–15% risk of progression, especially in the presence of persistent HPV16. Screening trials and vaccine evaluations with CIN2+ endpoints have documented the efficacy of the HPV test and a dramatic reduction in lesions in vaccinated cohorts, which was also confirmed for isolated CIN2. The most recent guidelines have progressively adopted a risk-based approach, which allows for active surveillance in young women or those seeking to conceive, while the WHO maintains a screen-and-treat model in resource-limited countries. **Conclusions:** CIN2 is not a lesion to be treated automatically, but rather a paradigmatic model for personalized management. Integrating epidemiological and clinical data, supported by biomarkers, allows for reducing overtreatment without compromising oncological safety.

## 1. Introduction

Persistent high-risk human papillomavirus (HPV) infection is the necessary etiological factor for the development of cervical cancer, a major cause of cancer incidence and mortality in women globally [1]. Secondary prevention, initially based on the Pap test and now increasingly based on the use of the HPV test, has led to a substantial reduction in the burden of the disease in countries with organized screening programs [2]. At the same time, the introduction of prophylactic vaccination has inaugurated a new era in primary prevention, with an already evident impact on the incidence of precancerous lesions [3].

Within the spectrum of HPV-related histological alterations, cervical intraepithelial neoplasia grade 2 (CIN2) occupies a controversial position. Classified as a high-grade squamous intraepithelial lesion (HSIL), CIN2 has traditionally been considered an indication for treatment, as it is part of the continuum leading to invasive carcinoma. However, numerous studies have demonstrated that CIN2 has peculiar characteristics: it is biologically unstable, associated with high interobserver diagnostic variability, and has a much higher rate of spontaneous regression than CIN3, especially in young women [4,5,6,7].

This biological instability has fueled a long-standing methodological tension. In large screening and vaccine evaluation trials, CIN2 has historically been aggregated with CIN3 in the combined CIN2+ endpoint. This choice responded to the need for diagnostic stability and statistical power, making the data more robust and suitable for supporting public health decisions [8,9]. The use of CIN2+ has demonstrated the efficacy of HPV testing and vaccination, but at the cost of obscuring the clinical and prognostic characteristics of CIN2 alone.

In recent years, with the consolidation of data from prospective clinical trials and the introduction of biomarkers (p16, Ki-67 dual stain, E6/E7 mRNA), the distinction between CIN2 and CIN3 has returned to the center of attention. The most recent international guidelines have progressively recognized that CIN2, unlike CIN3, can be managed conservatively in selected patients, favoring active surveillance in young women and those with reproductive desire [10,11,12,13,14].

In parallel, the impact of HPV vaccination has made this dichotomy even more evident: while the incidence of CIN2+ has plummeted in vaccinated cohorts, studies that have distinguished isolated CIN2 have highlighted that the reduction is particularly marked for this lesion, which is more unstable and strictly correlated to short-term HPV infections [15,16,17].

These findings have favored a paradigm shift in management, moving from an approach based on systematic excisional treatment to a risk-based management, in which active surveillance and the use of predictive biomarkers are increasingly considered safe alternatives to immediate treatment.

In this scenario, more refined and personalized management models have been developed, some of which—such as the risk-based management proposed by the American Society for Colposcopy and Cervical Pathology (ASCCP) in the United States—integrate predictive algorithms capable of estimating the risk of progression to CIN3+ based on multiple clinical and virological variables. In Europe, the European Society of Gynecological Oncology (ESGO), National Institute for Health and Care Excellence (NICE), and Italian GISCi guidelines also propose risk stratification criteria that include age, HPV genotype, cytology, colposcopic visibility, and immune status.

In light of these developments, the management of CIN2 can no longer be addressed with a single approach but must necessarily be based on solid evidence, up-to-date predictive tools and informed patient involvement in the decision-making process. Active surveillance, once considered a second-line option, is becoming a clinically valid option, supported by extensive efficacy and safety data.

This review aims to: Analyze the available literature on the natural history of CIN2, with particular attention to the rates of regression, persistence and progression of untreated CIN2 lesions. Identify the main prognostic factors that can guide therapeutic choice (age, HPV genotype, cytology, immune status). Compare the most authoritative international guidelines (ASCCP, ESGO, GISCi, WHO, NICE) on the management of CIN2 to assess consistency, operational differences, and practical implications for the management of CIN2 in the era of risk-based management.

## 2. Materials and Methods

### 2.1. Study Design

To prepare this review, an extensive literature search was conducted in the PubMed/MEDLINE and Scopus databases, complemented by a manual analysis of the bibliographies of selected articles (snowballing). The search considered articles published between 1990 and January 2025, in English or Italian, with available abstracts. Combinations of MeSH and free terms were used, such as “CIN2,” “cervical intraepithelial neoplasia grade 2,” “epidemiology,” “incidence,” “prevalence,” “screening,” “HPV vaccination,” “natural history,” “regression,” “progression,” “management,” “guidelines,” and “biomarkers.” Queries were adapted to each database to optimize search sensitivity.

#### 2.1.1. Inclusion Criteria

- Prospective or retrospective clinical studies with specific data on isolated CIN2 (incidence, regression, progression, biological predictors).
- Population-based studies and screening trials using the CIN2+ endpoint, including RCTs (NTCC, ATHENA, Nordic studies) and population-based cohorts (KPNC, HPV-IMPACT).
- Studies on the impact of HPV vaccination with CIN2 or CIN2+ outcomes.
- Guidelines and consensus documents from scientific societies and institutions (ASCCP, ACOG, ESGO/EFC, GISCi, Ministry of Health, NHS, WHO).
- Studies on biomarkers (p16, Ki-67 dual stain, E6/E7 mRNA, methylation) relevant to the diagnosis or management of CIN2.

#### 2.1.2. Exclusion Criteria

- Case reports and case series with <30 patients.
- Studies reporting exclusively on CIN3 without data on CIN2.
- Articles not accessible in full text and without abstracts.

The identified articles were evaluated in two stages. In an initial screening, based on the title and abstract, irrelevant contributions, case reports and case series with fewer than 30 patients, studies reporting exclusively CIN3 data without mentioning CIN2, were excluded. Subsequently, the full texts of the remaining articles were examined for relevant information on isolated CIN2 or the aggregate CIN2+ endpoint.

### 2.2. Selection and Categorization

For each article, the endpoint considered (CIN2 vs. CIN2+) was noted to clarify the difference in context.

The included studies were divided into three broad categories:

1. Those reporting specific data on isolated CIN2, such as prospective or retrospective clinical studies dedicated to its natural history, regression, progression, and biomarkers;
2. Those using the CIN2+ endpoint, typically screening trials, population-based cohorts, and studies on the impact of vaccination, in which CIN2 was aggregated with CIN3 for reasons of diagnostic stability and statistical power;
3. Consensus documents and international guidelines, including ASCCP, ACOG, ESGO/EFC, GISCi, Ministry of Health, NHS, and WHO.

### 2.3. Methodological Considerations

A crucial aspect of this review concerns the management of the methodological distinction between studies analyzing CIN2 as a standalone entity and studies reporting outcomes in aggregate form as CIN2+ (CIN2 and CIN3). The decision to include both types of studies was not accidental, but rather responds to complementary scientific and clinical needs.

On the one hand, isolated CIN2 today represents an entity of primary clinical interest. For these reasons, CIN2 is the focus of prospective and observational studies aimed at defining active surveillance strategies and identifying biomarkers useful for risk stratification (p16, Ki-67, E6/E7 mRNA, methylation testing). Analysis of CIN2 alone is therefore essential to understand the prognostic factors that guide daily clinical practice and to reduce the risk of overtreatment in low-risk patients [18].

On the other hand, studies using the CIN2+ category as an endpoint cannot be ignored. This methodological choice, widely used in large screening trials (e.g., NTCC, ATHENA, KPNC) and vaccination evaluation programs, responds to several needs. First, the use of CIN2+ increases the statistical power of analyses, allowing for the detection of significant differences in population-based studies with extended follow-up. Second, the histological diagnosis of CIN2 is characterized by high interobserver variability and less stability than CIN3; combining the two lesions in a single endpoint therefore reduces the risk of misclassification and ensures greater comparative robustness across studies and different cohorts. Finally, CIN2+ is the endpoint recognized at the regulatory level by international agencies (WHO, FDA, EMA) as a validated surrogate for cervical cancer, thus representing the gold standard for measuring the impact of prevention policies, particularly HPV vaccination.

The methodological choice to include both studies focused on isolated CIN2 and studies based on the CIN2+ endpoint responds to the need to provide a comprehensive overview of the topic. The former allow us to understand the natural history of the lesion and guide individual clinical decisions, while the latter provide solid data to describe the population epidemiology and measure the impact of preventive interventions. This integrated approach allows us to enhance both perspectives, while recognizing the intrinsic limitations: on the one hand, the diagnosis of CIN2 suffers from a known interobserver variability; on the other, aggregated endpoints may mask the clinical specificity of CIN2.

**Figure 1:**
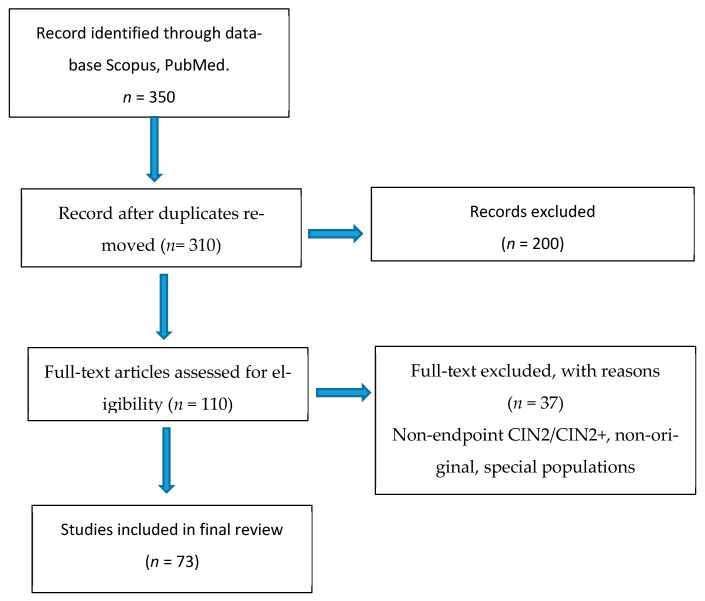
Study selection flow-chart based on PRISMA Style.

As this was a narrative review, a PRISMA protocol was not adopted, nor was a quantitative meta-analysis conducted; however, for transparency, a flow chart was based on the style of PRISMA protocol to illustrate the study selection process (Figure 1).

The objective was not to statistically synthesize the results, but rather to construct a critical and comparative synthesis of the available evidence, paying attention to the methodological differences between clinical trials and population-based studies and the implications of these differences for the management of CIN2 in clinical practice and public health (Supplementary Table S1).

## 3. Results

The search yielded a total of 350 articles, identified through major scientific databases (PubMed, Scopus). After removing duplicates (40) and an initial screening of titles and abstracts (200), 110 articles remained, fully assessed in full text. Of these, 73 were included in the final qualitative review. The analyzed corpus included prospective and retrospective observational studies, systematic reviews, and meta-analyses, as well as official international clinical guideline documents, selected to represent current management and the most up-to-date evidence on cervical intraepithelial neoplasia grade 2 (CIN2). Summary of included studies (*n* = 73).

### 3.1. Epidemiology

Estimating the prevalence of CIN2 is challenging because most data derive from aggregate CIN2+ endpoints. Historically, large screening trials and vaccine efficacy studies have adopted the composite CIN2+, which includes CIN2, CIN3, and more advanced forms, as the primary outcome. This approach has led to a large availability of data on CIN2+, but at the cost of limited specific knowledge of CIN2 alone. Screening studies (NTCC, ATHENA, KPNC) report CIN2+ prevalences between 0.5 and 1.5% [8,9,19].

Prospective studies that have clearly distinguished isolated CIN2 have documented relatively low incidences (0.2–0.4% in screening populations) and a peculiar biological behavior, characterized by a high propensity for spontaneous regression, especially in young women [10,11,13,14,17,20,21,22]. An international meta-analysis estimated that approximately 50% of CIN2 regress within two years, with rates that can exceed 60% in women under 25 years of age [21]. At the same time, the risk of progression to CIN3 or beyond has been found to be low, around 10–15%, especially in cases associated with persistent HPV16 infection [22].

With the advent of HPV vaccination, the incidence of CIN2+ has decreased dramatically in many populations. Large-scale studies in England, the United States and in global meta-analyses have confirmed significant declines, with reductions of up to 70–80% in fully vaccinated cohorts [15,16,23].

Australia, the first country to introduce a large-scale national vaccination program (since 2007), represents an emblematic case: within a few years of its launch, rapid and significant reductions in CIN2+ were observed in vaccinated young women, confirmed by subsequent national data linkage studies, even more in CIN2 cases, more instable [24,25,26,27].

Converging data emerge from health systems with robust registries: in the UK, the NHS England population-based study documented marked declines in high-grade lesions and invasive carcinoma in early vaccinated cohorts [16]; in the US, HPV-IMPACT surveillance shows a sustained decline in CIN2+ between 2008 and 2022, particularly pronounced in young women [23].

In summary, while studies with CIN2+ remain essential for describing epidemiological trends and the impact of public health interventions, investigations analyzing isolated CIN2 provide essential information for individual clinical management, providing a more realistic picture of its natural history. Critically integrating both perspectives is therefore a necessary step to fully understand the epidemiology of CIN2.

While epidemiological studies have clarified the overall impact of prevention on CIN2 and CIN2+, it remains essential to delve deeper into the natural history of isolated CIN2 to understand its true potential for regression or progression and more accurately define the clinical implications of its diagnosis.

### 3.2. Natural History of CIN2

Histologically classified as a high-grade squamous intraepithelial lesion (HSIL), CIN2 falls somewhere between CIN1, generally considered a low-risk and self-limiting lesion, and CIN3, unanimously interpreted as an immediate precursor of cervical cancer [4,28].

An element of complexity is represented by the poor diagnostic reproducibility. The ALTS study showed that interobserver agreement in the histological diagnosis of CIN2 is substantially lower than that of CIN3, with a significant rate of retrospective reclassification [5]. This aspect has led international consensus groups (Darragh TM, LAST Project 2012) to recommend the use of the p16 biomarker as an “adjudication” criterion to distinguish true CIN2 from reactive or borderline alterations [6].

The peculiarity of CIN2 lies in its biological instability. Since the 1990s, Ostör had highlighted how CIN2 did not constitute a homogeneous nosological entity, but rather a “point of equilibrium” between regression and progression, with a significant proportion of lesions destined to regress spontaneously [4]. Unlike CIN3, which shows an almost invariably persistent and progressive behavior, CIN2 is characterized by a range of possible outcomes: complete regression, stable persistence or progression towards a higher grade lesion.

The scientific literature analyzed for this review increasingly clearly highlights how CIN2 cannot be interpreted as a univocal lesion, but rather represents a biologically heterogeneous entity, whose clinical course is modulated by a multiplicity of factors, both viral and immunological, cytological and demographic.

#### 3.2.1. Spontaneous Regression

In observational cohorts including women with a histologically confirmed diagnosis of CIN2, the likelihood of spontaneous regression was surprisingly high. Significant rates of spontaneous regression of CIN2 have been reported in several cohorts. Tainio et al. conducted one of the largest meta-analyses available, documenting a 50% regression rate at 24 months, with peaks exceeding 65% in women <25 years of age [12]. Similar findings were confirmed by other studies, which highlighted the high likelihood of regression in young women with non-HPV 16/18 infection [10,11,14,29]. Lycke et al. also described a high rate of regression in the early stages of follow-up, although a progressively increased risk of progression was observed in cases observed beyond 24 months [22]. Other authors confirmed that lesions associated with low-grade cytology, satisfactory colposcopy, and negative biomarkers tend to regress more frequently [21,30,31,32,33].

Ehret et al. suggested that regular monitoring allows for the detection of most no regressing lesions, limiting the risk of progression to highly selected cases [13].

This trend has been confirmed in diverse settings, from Northern Europe to the United States, strengthening the hypothesis that in the presence of an efficient immune system and a physiological inflammatory response, many CIN2 lesions are able to resolve without the need for treatment.

The most recent meta-analyses have consolidated this finding, reporting an average regression rate of 50–60% in unselected populations. This is a percentage that is far from negligible, and has profoundly influenced the rethinking of clinical strategies in recent years. Not only that: even in women over 30 years of age, in the absence of other risk factors, regression is not a rare event, although less frequent than in younger age groups [12].

#### 3.2.2. Persistence

Alongside regression, lesion persistence should be considered, defined as the histological stability of CIN2 for a period exceeding 12 months. Persistence has been documented in approximately 25–30% of women included in major follow-up studies [34,35,36].

Although lesion persistence beyond 12–18 months has historically been considered an indicator of increased risk for progression, the literature demonstrates that persistence does not necessarily equate to progression. Prospective studies have documented spontaneous regressions even in cases defined as “persistent,” especially in young women: Moscicki et al. observed that a significant proportion of CIN2 cases regressed even after 24 months of follow-up, while the meta-analysis by Tainio et al. highlighted that approximately 40% of persistent CIN2 cases still regressed with prolonged observation [10,12]. Koeneman and colleagues confirmed the fluctuating nature of the lesion, characterized by phases of persistence followed by late regression [20].

From a clinical perspective, persistence takes on a different meaning depending on the context. In young women (<25 years), persistence alone does not automatically indicate treatment unless HPV16 or other risk factors are present. Conversely, in adult women (>25 years), international guidelines (ASCCP 2019; ESGO/EFC 2020; GISCi 2021) recommend treatment if the condition persists beyond 24 months to reduce the risk of progression. Finally, in immunocompromised women, persistence takes on even greater clinical relevance, with a higher risk of progression, making active surveillance less safe. These data emphasize that the management of CIN2 must be based not only on the presence or absence of persistence, but on an integrated assessment of age, viral genotype, and immune status, in a truly risk-based approach.

#### 3.2.3. Progression

The most concerning finding, and one that has historically justified systematic treatment, is the possibility of progression to high-grade lesions, particularly CIN3 and carcinoma in situ. The risk of progression to CIN3+ has been evaluated in several studies: Lycke et al. reported a rate of 33% with extended follow-up beyond two years, while Loopik et al. and Tainio et al. highlighted rates between 10 and 20%, especially in the presence of HPV 16, HSIL cytology, or viral persistence [11,12,22].

Other studies confirm that progression is more frequent in patients with persistent high-risk genotypes, unsatisfactory colposcopy, or in women over 30 [20,37,38,39]. Del Prete et al., in a large Italian cohort, observed that only a minority of low-risk cases progress to severe lesions [40].

These numbers, although smaller than the general population, justify attention and caution in recommending observation alone, which must always be supported by rigorous follow-up and clear selection criteria. Some authors also emphasized the poor diagnostic reproducibility of CIN2, suggesting that a portion of lesions classified as such may actually be borderline histological artifacts [4,5]. These data emphasize the need for a careful balance between active surveillance and therapeutic intervention, particularly when the risk of progression is accompanied by unfavorable prognostic factors (Table 1) [18].

### 3.3. Prognostic Factors

The prognostic factors identified in the various studies represent one of the most significant contributions of this review. The analysis of prognostic factors has allowed us to more precisely identify the patient subgroups in which an active surveillance strategy is safe and effective.

#### 3.3.1. Age

Patient age remains the primary discriminating criterion: women under 30, particularly if HPV-negative or carrying viral genotypes other than 16/18, show significantly more favorable clinical behavior [11,14,21,31]. This finding is so well-established that it has been adopted by almost all international guidelines to identify patients eligible for active surveillance. Conversely, age over 30, especially if associated with HSIL cytology and persistent high-risk HPV infection, is associated with a poorer prognosis, with a higher incidence of persistence or progression.

#### 3.3.2. HPV Genotype

A central element in the prognostic assessment is the HPV genotype. HPV 16, in particular, remains the main predictor of progression, not only due to its affinity with the cervical transformation zone, but also due to its ability to induce genomic instability and resistance to immune, while the presence of genotypes other than 16/18 is associated with more favorable outcomes [41,42,43]. HPV 18, although less frequently associated with CIN2, is often linked to glandular lesions and adenocarcinoma in situ, representing another warning element. The presence of these genotypes, especially if persistent, represents a condition in which surveillance becomes less reliable and treatment gains preferential indication.

#### 3.3.3. Biopsy-Associated Cytology

Biopsy-associated cytology plays a key prognostic role. The presence of high-grade cytology (HSIL) at diagnosis is associated with a significantly increased risk of progression, so much so that many guidelines consider its presence alone sufficient to recommend treatment. Conversely, low-grade cytology (LSIL) or a simple ASC-US, in the presence of a CIN2 biopsy, suggests a less aggressive lesion and more prone to regression.

Biopsy-associated cytology provides an additional element of evaluation: a diagnosis of HSIL on a Pap test, in conjunction with a CIN2 biopsy, represents a strong signal of high risk [31,38]. Conversely, the presence of low-grade cytology (ASC-US or LSIL) suggests a less biologically aggressive lesion, often worthy of observation [32,36,37].

In some cases, discordance between cytology and histology may even indicate an overestimation of the lesion by the biopsy, as occurs in the presence of reactive inflammation or technical artifacts.

#### 3.3.4. Immune Status

In addition to age, HPV, and cytology, other clinically relevant factors emerge. The patient’s immune status is a critical variable: immunocompromised women, particularly those with HIV or on chronic immunosuppressive therapy, show a greater tendency towards progression, with significantly higher rates than the general population [44,45,46,47]. In these cases, active surveillance is discouraged by most guidelines, which recommend treatment as the preferred option, even for intermediate-grade lesions.

#### 3.3.5. Cigarette Smoking

Cigarette smoking has been identified as an independent risk factor for the persistence and progression of CIN2 [20]. Toxic substances contained in smoke alter the cervical microenvironment, reduce the local immune response, and facilitate the integration of the viral genome into host cells. For this reason, even in young women, the presence of an active smoking habit leads to a more cautious clinical approach.

#### 3.3.6. HPV Vaccination

Finally, it is worth highlighting the emerging value of HPV vaccination as not only a preventive tool but also an adjuvant therapeutic tool. The potential of post-treatment vaccination has been confirmed by numerous authors, who demonstrated a significant reduction in the risk of recurrence after LEEP or conization, even in women vaccinated for the first time in adulthood [48,49,50]. These data were confirmed in American registries following the introduction of the quadrivalent vaccine [23].

Australia represents the first and most emblematic example of the success of population-wide HPV vaccination. The national program, launched in 2007 with the quadrivalent vaccine and subsequently extended to men and the nonavalent vaccine, has produced an unprecedented reduction in cervical precancerous lesions in just a few years. As early as 2011, Brotherton et al. documented a significant decline in CIN2+ diagnoses in vaccinated young women, compared to unvaccinated cohorts [24]. These data were confirmed and expanded by subsequent national data linkage studies, which showed marked and progressive reductions in CIN2+ across all younger age groups [25].

The Australian experience had a paradigmatic impact not only due to the rapidity of the observed reduction, but also due to the depth of the epidemiological transformation: in just a few years, the prevalence of vaccine-related HPV genotypes collapsed to levels close to zero [51,52], suggesting a direct and indirect effect (herd protection) on viral circulation. Subsequent evidence has allowed us to estimate that the country is on track to become the first in the world to virtually eliminate cervical cancer by 2035, thanks to the combination of vaccination and organized screening [53].

In summary, Australia demonstrates how a high-coverage vaccination program can rapidly lead to a decline in CIN2+ and probably to a greater extent in isolated CIN2, as demonstrated by Swedish national data showing a more rapid decline of early lesions in vaccinated cohorts [54]. This model provides a benchmark for global cervical cancer elimination strategies [55].

### 3.4. Biomarkers

In addition to cytology, HPV genotyping and colposcopy, several molecular biomarkers have also acquired a potentially useful role in stratifying the risk of progression of CIN2 lesions. p16 expression, recommended for diagnostic confirmation [6], correlates with a higher risk of persistence, while p16/Ki-67 dual stain positivity is associated with an increased risk of progression [30,35,37,56]. A similar consideration applies to viral mRNA (E6/E7) molecular tests, which have shown good specificity but remain scarcely available in routine practice [33]. Recent studies have also explored the role of gene methylation (FAM19A4/miR124-2), with promising results in prognostic stratification [57,58,59].

However, the clinical use of these tests is limited by several factors: uneven availability across healthcare settings, interlaboratory variability in immunohistochemical interpretation, and the lack of international standardization regarding thresholds and reading methods. Within the context of new risk-based management models (e.g., ASCCP Risk-Based Management), these biomarkers may serve as complementary, rather than substitutive, tools to be applied in selected clinical situations, pending broader validation and technical harmonization.

### 3.5. Guidelines

The evolution of knowledge on the natural history of CIN2 has also been progressively reflected in the main international guidelines, which have gradually relaxed the indication for immediate treatment in favor of a more individualized approach.

In the United States, the 2019 ASCCP guidelines represented a turning point, introducing the paradigm of risk-based management [60]. In this model, clinical decisions are no longer based solely on histological findings, but on a personalized estimate of the risk of persistent or progressive high-grade lesions, calculated by integrating age, clinical history and virologic results. In particular, for young women (<25 years) and those seeking to conceive, active surveillance for CIN2 is considered a safe option, provided that p16-negative lesions are excluded and rigorous follow-up is ensured [19]. The ACOG has incorporated these recommendations into two practice advisories (2020 and 2021), ACOG Advisory 2020 and ACOG Screening Update 2021, confirming their validity even in the broader context of reproductive health [61,62].

In Europe, the ESGO/EFC (European Federation for Colposcopy) guidelines (2020) maintain CIN2 distinct from CIN3 and have adopted a similar position, recommending a conservative approach in young women and favoring treatment for patients over 30 years of age or in the presence of persistent HPV16/18 infection [63]. The European guidelines have also emphasized the role of biomarkers: p16 as a diagnostic tool to reduce histological variability and the dual stain p16/Ki-67 as an aid in risk stratification. The NICE guidelines (UK) also allow conservative management of CIN2 in patients <25 years of age or with reproductive desire, provided that rigorous colposcopic follow-up is guaranteed [64]. The United Kingdom has strengthened this approach through the histopathological guidelines of the NHS Cervical Screening Program (NHS CSP, 2024) which recommend the routine use of p16 when HSIL is suspected but histologically uncertain, with the explicit aim of reducing the risk of overdiagnosis of CIN2 [65,66]. In parallel, active surveillance experiences in cohorts of young women, conducted in Nordic countries and the United Kingdom, have provided solid safety evidence, showing high regression rates and a low risk of progression, elements that have strengthened the legitimacy of conservative management.

In the Italian context, the recommendations of the GISCi (Italian Cervical Cancer Screening Group) and the documents of the Ministry of Health confirm the European approach [67,68]. Italian recommendations state that CIN2 can be managed with active surveillance in women <25 years of age and in those with reproductive desire, provided that high-quality colposcopy and structured follow-up are available. In persistent or progressive p16-positive cases, excisional treatment remains indicated. Therefore, even in Italy, there has been a shift from a “treat all CIN2/3” approach to a clinically individualized model.

Globally, the WHO guidelines (2021, 2024) published in 2021, with updates in 2024, maintain a more pragmatic and public health-oriented approach in resource-limited settings [69,70]. In these settings, where the possibility of structured follow-up is limited and the risk of loss to follow-up is high, the recommendation is to directly treat all HSIL (CIN2/3) according to a “screen-and-treat” model, favoring ablation or immediate excision. The priority remains the reduction of the incidence of invasive cancer and the practical implementation of HPV-based screening programs. However, in the sections dedicated to high-income settings, the WHO recognizes the validity of active surveillance of CIN2 in selected populations, in line with ASCCP and European recommendations (Table 2).

Active Surveillance vs. Treatment

The clinical balance point is common to all documents:

▪ Active surveillance: an appropriate option for young women and those with reproductive desire, especially in the absence of persistent HPV16 and when the lesion is p16-confirmed but clinically “stable.” The goal is to avoid overtreatment and reduce the obstetric risks associated with excisional procedures (ASCCP; ESGO/EFC; GISCi; NICE/NHS).
▪ Excisional treatment: indicated in cases of persistence (generally more than 24 months in adults), the presence of high-risk factors (persistent HPV16, immunocompromised), significant diagnostic discrepancies, or cytological/histological progression. Attention to reproductive implications remains central: many guidelines recommend minimizing the volume and depth of excision when indicated, and involving patients in a shared decision-making process.

These elements explain why the most recent guidelines (ASCCP, ESGO, GISCi) have introduced the possibility of active surveillance for CIN2 in young women or those with reproductive desire [60,63,67]. At the same time, they confirm the need to treat higher-risk cases, especially in the presence of persistent HPV16 infection or unfavorable biomarkers (Table 3).

## 4. Discussion

The management of CIN2 represents one of the most emblematic examples of how modern medicine is evolving from a rigidly interventional approach to a model based on personalized risk assessment. For a long time, the histological diagnosis of CIN2 was considered an automatic indication for treatment, based on the belief that it was an obligatory precursor to cervical cancer. However, the evidence accumulated over the past two decades has demonstrated that this view was reductive: CIN2 is a biologically heterogeneous lesion, capable of spontaneous regressing in a significant proportion of cases, but also of progressing in a minority of patients, particularly when associated with persistent HPV16 or immunocompromised conditions.

This new awareness has radically changed the clinical perspective. Today, the challenge is no longer “to treat or not to treat,” but rather to identify as precisely as possible cases at risk of progression and distinguish them from those that can be managed with active surveillance. In this sense, CIN2 has become a paradigm of personalized medicine, in which therapeutic decisions depend on a variety of factors: virological characteristics, age, immune status, reproductive desire, and the availability of advanced diagnostic tools.

An important aspect concerns the way data were collected and analyzed. For many years, most studies used the CIN2+ endpoint; that is, they evaluated CIN2 and CIN3 diagnoses together. This choice made epidemiological analyses more robust and comparable, and allowed the impact of vaccination to be clearly measured. However, it also obscured the natural history of CIN2 alone. Therefore, today it is necessary to integrate both perspectives: population-based studies using CIN2+, which are essential for public health, and those analyzing isolated CIN2, which are essential for guiding individual clinical decisions.

On a clinical level, the debate has been strongly influenced by the growing awareness of the obstetric risks associated with excisional treatments [71,72,73]. This awareness has prompted guidelines to propose active surveillance pathways based on structured follow-ups, especially for young patients or those seeking to conceive. In this context, biomarkers are playing an increasingly central role. The use of p16, the p16/Ki-67 dual stain, mRNA tests, and epigenetic markers offer tools to reduce diagnostic subjectivity and more accurately stratify developmental risk, contributing to more informed and confident clinical decisions.

The introduction and widespread use of HPV vaccination have further changed the landscape. The dramatic reduction in CIN2+ in vaccinated populations, which occurred more rapidly for CIN2 than for CIN3, demonstrates the impact of primary prevention not only on cancer incidence but also on the biology and epidemiology of precancerous lesions. This shift requires organizational rethinking: in a context of lower prevalence, adopting highly specific screening and triage pathways is essential to avoid unnecessary treatments and overdiagnosis. The introduction of primary HPV screening and more sophisticated triage reduces the number of colposcopies and unnecessary treatments, while programs like the NHS CSP have introduced extended booster intervals for HPV-negative women. This makes the system more efficient, but requires constant auditing and quality control throughout the diagnostic chain.

At the same time, the emergence of post-treatment adjuvant vaccination opens new perspectives for integrating primary and secondary prevention, with potential benefits for reducing recurrences.

Finally, the discussion on CIN2 reflects a broader aspect of contemporary medicine: the need for an approach that we might define as dynamic and contextual, capable of balancing cancer prevention, reducing overtreatment, and improving quality of life. International guidelines are gradually converging toward this vision, but their implementation remains conditioned by organizational differences and the resources available in different healthcare systems. Indeed, the main differences concern the context of application: in the United States and Europe, a risk-based approach prevails, integrating biomarkers (p16, dual-stain, mRNA) and predictive tools to personalize the decision; in Italy, this approach has been adopted and adapted within organized screening programs; in low- and middle-income countries, WHO recommendations favor a pragmatic screen-and-treat model, aimed at reducing loss to follow-up and mortality. Overall, the management of CIN2 today represents a paradigmatic example of the integration of population epidemiology and individual clinical practice, where the healthcare context and available resources substantially influence the recommended strategies.

CIN2, therefore, is not only a gynecological oncology problem, but also a testing ground for management models that integrate epidemiology, biology, public health, and precision medicine.

## 5. Conclusions

CIN2 is not a lesion to be treated automatically: integrating epidemiological data, natural history and biomarkers is the key to risk-based management that reduces overtreatment and maintains oncological safety.

## Figures and Tables

**Table 1:** Some references cited in the study about Regression and Progression of CIN2/CIN3.

Reference	Study Design	Population	*N*	Follow-Up	Regression Rate	Progression Rate	Notes/Relevance
Ostör, A.G., 1993 [4]	Critical review	CIN1-3	NA	Various	60%	10%	Supports 50–70% regression, 10–15% progression
Moscicki, A.B., 2010 [10]	Prospective cohort	Adolescents/young women with CIN2	237	36 mo	68%	12%	Regression higher in young women
Loopik, D.L., 2016 [11]	Cohort	Women <25 years with CIN2	112	24 mo	65%	13%	Age <25 predictive of regression
Tainio, K., 2018 [12]	Systematic review & meta-analysis	CIN2 under surveillance	1635	24–36 mo	55–63%	11–14%	Aggregated data supporting summary figures
Ehret, A., 2023 [13]	Cohort	Women <25 years with high-grade lesions	89	24 mo	62%	10%	Young age and lesion size influence regression
Skorstengaard, M., 2020 [14]	Cohort	CIN2 women, Denmark	275	24 mo	58%	12%	Supports conservative management evidence
Silver, M.I., 2018 [21]	Cohort	Women 21–39 years, CIN2	270	18 mo	57%	13%	Regression consistent with 50–70% range

**Table 2:** Guidelines for the management of CIN2 (international comparison).

**ASCCP (USA)** [60]	2019	p16 as adjudication (LAST); HPV testing for follow-up	Recommended observation: colposcopy + HPV testing every 6–12 months up to 24 months	Excisional treatment preferred; observation acceptable if reproductive desire and visible transformation zone	Risk-based model: personalized management based on the estimated risk of CIN3+
**ACOG (USA)** [61]	2020	Accepts use of p16	Same as ASCCP	Same as ASCCP	Joins the ASCCP 2019
**ESGO/EFC (Europe)** [63]	2020	Mandatory use of p16 for all CIN2; optional Ki-67/mRNA	Active surveillance is recommended; follow-up with colposcopy + HPV testing every 6–12 months	Treatment recommended if persistence >24 months or HPV 16 persistent; surveillance possible in selected cases	Conservative and personalized approach, strong role of biomarkers
**NHS CSP (UK)** [65]	2024	p16 recommended as triage	Recommended observation in patients <25 years of age or with reproductive desire	Treatment or surveillance if justified	Emphasis on auditing and overtreatment reduction
**GISCi (Italy)** [67]	2021	p16 mandatory; dual stain in reference centers	Active surveillance in <25 or in women with reproductive desire	Treatment or follow-up if compliance is high	Aligned with ESGO, strong use of biomarkers
**Ministry of Health (Italy)** [68]	2024	Confirm p16 use; HPV test for follow-up	Recommended observation	Standard treatment, observation in selected cases	National organized screening policy
**WHO** [69,70]	2021–24	Not recommended for LMIC; focus on HPV testing	Not distinguished from those ≥25 years	Not distinguished from <25 years	Screen-and-treat approach: immediate treatment of all HSIL (CIN2/3) without histological confirmation in low-income countries

**Table 3:** Management of CIN2: yesterday (pre-vaccine) vs. today (risk-based).

Aspect	Yesterday—Pre-Vaccine/Traditional Approach	Today—Post-Vaccine Era/Risk-Based Management
**Endpoint reported**	Prevalence/incidence often in CIN2+ for statistical power and greater diagnostic reproducibility [2,3,4,5].	In clinical trials, CIN2 is distinct from CIN3; in screening/vaccination, the CIN2+ public health endpoint remains [8,9,15,16,17].
**Natural history**	CIN2 frequently treated as “in need of treatment” HSIL. Historical data: prevalence ~0.2–0.4% [4,5].	High regression of CIN2 (≈40–70% in young women), rare progression; basis for active surveillance [10,14,20,21,22].
**Key findings**	Trial/registers: NTCC, ATHENA, KPNC [8,9,19].	Prospective studies/meta-analysis on isolated CIN2: Moscicki, Loopik, Tainio, Koeneman, Bruno, Lycke [10,11,12,18,20,22].
**Biomarkers**	Limited/heterogeneous use (p16 sometimes ancillary).	Triage p16/Ki 67, HPV mRNA E6/E7 and other markers to estimate regression/persistence and select who to monitor [18,35,37,38,56].
**Management strategy**	Prevalence of excisional treatment for HSIL.	Risk-based management: selective active surveillance (especially <25–30 years/reproductive desire); treatment if high risk or persistent [60,61,62].
**Impact of vaccination**	Not available / irrelevant.	Significant decline in CIN2+ at population level (−51% global meta-analysis; up to −79% 20–24 years USA; −33% Norway) and marked reduction in isolated CIN2 in vaccinated women (~0.2% vs. ~1%) [15,16,23].
**Main purpose**	Demonstrate test performance and screening coverage.	Integrating epidemiology and clinical practice: measuring population impact (CIN2+) and optimizing individual decisions (CIN2).

## Data Availability

No new data were created or analyzed in this study. Data sharing is not applicable to this article.

## Supplementary Materials

The following supporting information can be downloaded at: https://www.mdpi.com/article/10.3390/diagnostics15192512/s1, Table S1: Overview of the studies included in the review and their main characteristics.
