# Supplementary material for: CIN2 in the Era of Risk-Based Management and HPV Vaccination: Epidemiology, Natural History and Guidelines
Source: Diagnostics (Basel). 2025 Oct 2;15(19):2512. doi: 10.3390/diagnostics15192512 (PMC12524061; doi:10.3390/diagnostics15192512)
Supplement: Supplementary file 1 [file diagnostics-15-02512-s001.zip › diagnostics-3880510-supplementary.pdf]

Supplementary Table S1. Overview of the studies included in the review and their main characteristics.

N°	Name, Year	Type of Study	Country	Population	Follow-up	Outcome	Short note	Endpoint
1	Bosch FX et al., 2002	Review	International	N/A	N/A	HPV causes cervical cancer	Causal relationship between HPV and cervical cancer	CIN2+
2	Ronco G et al., 2014	RCT	Europe	Women in 4 European countries	Variable, follow-up years	Efficacy of HPV-based screening	HPV screening reduces invasive cancer	CIN2+
3	Drolet M et al., 2015	Meta-analysis	Multiple countries	Population-level data	N/A	Population-level impact of HPV vaccination	Direct effects and herd immunity	CIN2+
4	Ostör AG, 1993	Review	UK	N/A	N/A	Natural history of CIN	Historical regression/progression	CIN2+ (CIN2)
5	Stoler MH et al., 2001	Observational study	USA	Women with ASCUS/LSIL cytology	N/A	Interobserver reproducibility	Cytology/histology variability	CIN2+ (CIN2)
6	Darragh TM et al., 2013	Consensus statement	USA	N/A	N/A	LAST project guidelines	Standard p16 for HSIL/CIN2	CIN2+ (CIN2)
7	Bruno MT et al., 2022	Cohort study	Italy	Women with CIN3 and biopsy-cone interval >11 weeks	Variable	Spontaneous regression	CIN3 regression data*	CIN3
8	Wright TC et al., 2015	RCT	USA	Women screened for cervical cancer	End of ATHENA study	HPV primary screening efficacy	Primary HPV > cytology	CIN2+
9	Ronco G et al., 2010	RCT	Italy	Women in NTCC study	Variable	Detection of CIN and invasive cancer	Superior to the Pap test	CIN2+
10	Moscicki AB et al., 2010	Cohort study	USA	Adolescents and young women with CIN2	Variable	Regression and risk factors	High regression <25 years	CIN2
11	Loopik DL et al., 2016	Cohort study	Netherlands	Women <25 with CIN2	Variable	Regression and progression predictors	High regression	CIN2
12	Tainio K et al., 2018	Systematic review & Meta-analysis	International	Women with untreated CIN2	Up to 24 months	Natural course under surveillance	Regression of 50–60%, Progression of 10–15%	CIN2
13	Ehret A et al., 2023	Cohort study	Germany	Women <25 with high-grade CIN	Variable	Regression rate	Follow-up regressions	CIN2+
14	Skorstengaard M et al., 2020	Cohort study	Denmark	Women with CIN2	Variable	Conservative management outcomes	Surveillance 23–29 years	CIN2

15	Drolet M et al., 2021	Modeling study	Low- & middle-income countries	Population-level	N/A	Optimal HPV vaccination strategies	HPV vaccinations strategies	CIN2+
16	Falcaro M et al., 2021	Observational study	UK	Women in national registry	Variable	Impact of HPV vaccination	Marked reductions in HG/cancer	CIN2+
17	Kyrgiou M et al., 2025	Consensus statement	Europe	Women with CIN2	N/A	Active surveillance recommendations	CIN2 active surveillance	CIN2
18	Bruno MT et al., 2020	Cohort study	Italy	Women with CIN2 p16+	Variable	Conservative management outcomes	Conservative management p16+	CIN2
19	Perkins RB et al., 2021	Guideline summary	USA	N/A	N/A	Screening and management	Screening	CIN2+
20	Koeneman MM et al., 2019	Cohort study	Netherlands	Women with high-risk HPV CIN2	Variable	Prognostic factors for regression	Regression/progression paths	CIN2
21	Silver MI et al., 2018	Cohort study	USA	Women 21-39 with CIN2	18 months	Clinical outcomes	HPV 16 persistent ↑ risk	CIN2
22	Lycke KD et al., 2023	Population-based cohort	Denmark	Women with CIN2	Variable	Clinical course	Real-world regression/persistence	CIN2
23	Gargano JW et al., 2025	Surveillance study	USA	HPV-IMPACT project	2008-2022	Trends in precancers	HPV surveillance	CIN2+
24	Brotherton JM et al., 2011	Ecological study	Australia	Women in Victoria	Early post-vaccination	HPV vaccination effect	First vaccination impact	CIN2+
25	Gertig DM et al., 2013	Data linkage study	Australia	Population-based	Variable	Impact on cervical abnormalities	Post-vaccine reduction	CIN2+
26	Baldur-Felskov B et al., 2014	Observational study	Denmark	Danish women	Pre- and post-vaccination	Incidence of cervical lesions	Significative decline	CIN2+
27	Sand FL et al., 2020	Cohort study	Denmark	Women post-conization	Variable	CIN2+ risk by HPV vaccination	CIN2+ risk after conization/vaccination	CIN2/3
28	Bruno MT et al., 2024	Cohort study	Italy	Women <30 with CIN3	Variable	Association with HPV16/18	HPV16/18 and CIN2 in women < 30 years old	CIN2
29	Reuschenbach M et al., 2022	Retrospective analysis	Germany	Women 18-45	Variable	Burden of CIN2+ and conizations	Epidemiological trends	CIN2+
30	Wentzensen N et al., 2019	Clinical evaluation	USA	Women in organized screening	Variable	HPV p16/Ki-67 dual stain performance	HPV triage with p16/Ki67	CIN2+
31	Massad LS et al., 2013	Consensus guideline	USA	N/A	N/A	Management of abnormal screening	Management anormal screening	CIN2+
32	Wentzensen N et al.,	Diagnostic study	USA	HPV+ women	Variable	p16/Ki-67 dual stain	P16/Ki67 for CIN2	CIN2+

	2015							
33	Bruno MT et al., 2022	Cohort study	Italy	Women with CIN2 and hr-HPV+	Variable	Negative E6/E7 mRNA as regression predictor	negative mRNA → regression	CIN2
34	Galgano MT et al., 2010	Diagnostic study	USA	Cervical biopsy samples	N/A	Biomarker use in diagnosis	Biomarkers	CIN2+
35	Wentzensen N et al., 2012	Diagnostic study	USA	Colposcopy referral population	Variable	p16/Ki-67 immunostaining performance	p16/Ki67 performance	CIN2+
36	Arbyn M et al., 2017	Systematic review & meta-analysis	International	Women with incomplete excision	Variable	Predictor of treatment failure	Positive margins and relapses	CIN2+
37	Wright TC et al., 2017	Sub-study RCT	USA	ATHENA trial participants	Variable	Triaging HPV+ women	p16/Ki67 dual stain	CIN2+
38	Clarke MA et al., 2024	Guideline	USA	HPV+ women	N/A	p16/Ki-67 dual stain recommendations	P16/Ki67 dual stain usage	CIN2+
39	Bruno MT et al., 2024	Retrospective cohort	Italy	Women undergoing LEEP for CIN3	18 months	HPV16 persistence and CIN2+ relapse	Relapses and CIN2+	CIN2+
40	Prete RD et al., 2019	Retrospective study	Italy	Women tested for HPV	Variable	Impact of PCR method change	Italian population data	CIN2+
41	Schiffman M et al., 2009	Review	International	N/A	N/A	Weakly carcinogenic HPV types	HPV classification	CIN2+
42	Katki HA et al., 2013	Cohort study	USA	Women with ASC-US Pap	5 years	CIN3+ risk	Long-term estimates	CIN2+
43	Schiffman M et al., 2011	Review	International	N/A	N/A	HPV testing in prevention	HPV testing prevention	CIN2+
44	Strickler HD et al., 2005	Cohort study	USA	HIV+ women	Variable	HPV natural history and reactivation	Persistence/progression in HIV	CIN2+
45	Denslow SA et al., 2014	Systematic review	Global	HIV+ women	Variable	Incidence and progression of cervical lesions	More frequent progression in HIV	CIN2+
46	Clifford GM et al., 2006	Meta-analysis	International	HIV+ women	Variable	HPV types in HIV	Risk ↑ in immunocompromised patients	CIN2+
47	Abraham AG et al., 2013	Prospective cohort	North America	HIV+ women	Variable	Invasive cervical cancer risk	Invasive cancer risk in HIV+ patients	CIN2+
48	Del Pino M et al., 2020	Cohort study	Spain	Women with CIN post-conization	Variable	HPV vaccination as adjuvant	Reduces relapse	CIN2+
49	Garland SM et al.,	RCT post-hoc	International	Women post-surgery	Variable	Recurrent high-grade CIN	Reduces post-LEEP relapses	CIN2+

	2016	analysis				prevention		
50	Kechagias KS et al., 2022	Systematic review & meta-analysis	International	Women post-surgery	Variable	HPV vaccination impact	Reduction in relapses ~65%	CIN2+
51	Machalek DA et al., 2018	Cross-sectional	Australia	18-35-year-old women	9 years post-vaccination	HPV type prevalence	Genotypes after vaccine	CIN2+
52	Drolet M et al., 2019	Systematic review & meta-analysis	Global	General population	Variable	HPV vaccination impact	Reductions of up to 70–80%	CIN2+
53	Hall MT et al., 2019	Modelling study	Australia	National population projections	Projected until elimination	Cervical cancer elimination timeframe	Impact on incidence	CIN2+
54	Lei J et al., 2020	Cohort study	Sweden	Vaccinated vs unvaccinated women	Up to 10 years	Risk of invasive cervical cancer	HPV vaccination significantly reduces invasive cervical cancer risk	CIN2+
55	Garland SM et al., 2016	Systematic review	Global	10 years of vaccine data	Up to 10 years	Vaccine effectiveness	Ten-year summary	CIN2+
56	Bruno MT et al., 2023	Observational	Italy	Elderly women with ASC-US	Not specified	Triage performance	p16/Ki67 in women HPV+	CIN2+
57	De Strooper LMA et al., 2016	Validation study	Netherlands	HPV-positive women	Not specified	Methylation test performance	Methylation test	CIN2+
58	Verhoef VM et al., 2014	Randomized controlled trial	Netherlands	HPV-positive self-samples	Not specified	Triage performance	Methylation vs cytology	CIN2+
59	Luttmer R et al., 2016	Comparative study	Netherlands	High-risk HPV-positive women	Not specified	Test performance	Methylation performance HPV+	CIN2+
60	Perkins RB et al., 2020	Consensus guideline	USA	Clinical population	Risk-based management	Management recommendations	Active, risk-based surveillance	CIN2+ (CIN2)
61	ACOG, 2020	Practice advisory	USA	Clinical population	Not specified	Screening management	Screening management	CIN2+ (CIN2)
62	ACOG, 2021	Practice bulletin	USA	Clinical population	Not specified	Screening	Cervical screening	CIN2+ (CIN2)
63	Kyrgiou M et al., 2020	Position paper	Europe	General population	Not specified	Screening recommendations	European recommendations	CIN2+ (CIN2)
64	NICE, 2024	Guideline summary	UK	General population	Not specified	Screening	Primary HPV, triage	CIN2+ (CIN2)
65	NHS CSP, 2024	National guideline	UK	Screening population	Not specified	Screening and colposcopy	Extended HPV ranges–	CIN2+ (CIN2)

66	NHS England, 2025	Policy update	UK	National population	Not specified	Screening rollout	Personalized screening	CIN2+ (CIN2)
67	GISCi, 2021	National guideline	Italy	CIN2 patients	Not specified	Management recommendations	CIN2+ (CIN2)	CIN2+ (CIN2)
68	Ministry of Health, 2024	National guideline	Italy	CIN patients	Not specified	Screening & biomarker guidance	CIN2+ (CIN2)	CIN2+ (CIN2)
69	WHO, 2021	Guideline	Global	Women eligible for screening	Not specified	Screening & treatment	CIN2+ (CIN2)	CIN2+ (CIN2)
70	WHO, 2024	Guidance	Global	HPV-positive women	Not specified	Triage with dual stain	CIN2+(CIN2)	CIN2+(CIN2)
71	Kyrgiou M et al., 2006	Systematic review & meta-analysis	Global	Women post-conservative treatment	Not specified	Obstetric outcomes	CIN2+	CIN2+
72	Arbyn M et al., 2008	Meta-analysis	Global	Women treated for CIN	Not specified	Adverse pregnancy outcomes	CIN2+ (CIN2)	CIN2+ (CIN2)
73	Kyrgiou M et al., 2017	Cochrane systematic review	Global	Women with CIN or early invasive disease	Not specified	Obstetric outcomes	CIN2+ (CIN2)	CIN2+ (CIN2)

Note: * The inclusion of Bruno et al. (2022), which focuses on CIN3 regression, provides a reference for comparison with CIN2 regression.
